# Surface Properties of the Hydrogen–Titanium System

**DOI:** 10.1021/acs.jpcc.1c08635

**Published:** 2021-11-09

**Authors:** Emanuel Billeter, Zbigniew Łodziana, Andreas Borgschulte

**Affiliations:** † Laboratory for Advanced Analytical Technologies, Empa—Swiss Federal Laboratories for Materials Science and Technology, Überlandstrasse 129, CH-8600 Dübendorf, Switzerland; ‡ Department of Chemistry, University of Zurich, Winterthurerstrasse 190, CH-8057 Zürich, Switzerland; § Institute of Nuclear Physics, Polish Academy of Sciences, PL-31342 Krakow, Poland

## Abstract

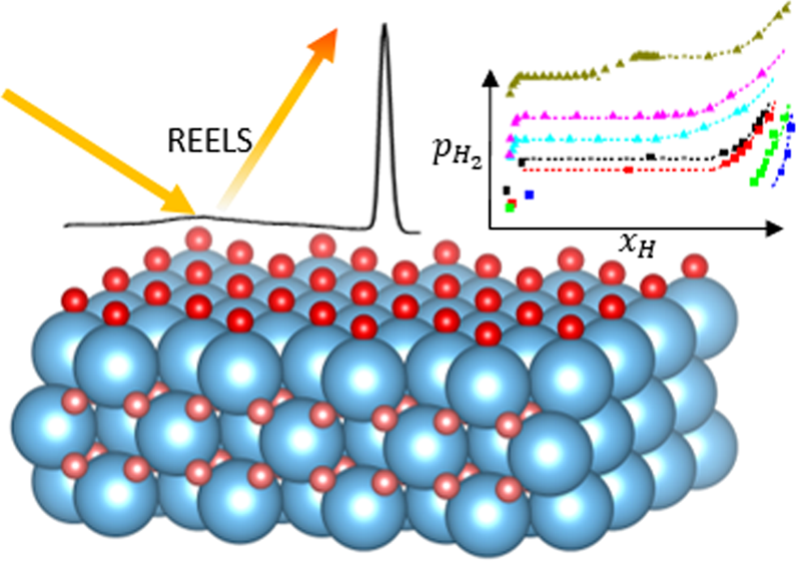

Titanium is an excellent getter material, catalyzes gas–solid reactions such as hydrogen absorption in lightweight metal hydrides and complex metal hydrides and has recently been shown as a potential ammonia synthesis catalyst. However, knowledge of the surface properties of this metal is limited when it absorbs large quantities of hydrogen at operation conditions. Both the conceptual description of such a surface as well as the experimental determination of surface hydrogen concentration on hydride-forming metals is challenging due to the dynamic bulk properties and the incompatibility of traditional surface science methods with the hydrogen pressure needed to form the metal hydride, respectively. In this paper, the surface pressure-composition isotherms of the titanium–hydrogen system are measured by operando reflecting electron energy loss spectroscopy (REELS). The titanium thin films were deposited on and hydrogenated through a palladium membrane, which provides an atomic hydrogen source under ultrahigh vacuum conditions. The measurements are supported by density functional theory calculations providing a complete picture of the hydrogen-deficient surface of TiH_2_ being the basis of its high catalytic activity.

## 1 Introduction

The widespread introduction of renewable hydrogen is still hampered by the difficulty of storing it efficiently at high volumetric and gravimetric density.^1^ The current technical solutions of storage as a gas under high pressure or as a liquid at very low temperatures are associated with limited storage density, efficiency, and/or safety issues.^2,3^

The catalytic conversion of H_2_ into energy-rich small molecules like CH_4_ or NH_3_ is a different strategy to store renewable energy. Here, storage is straightforward, and demand for research and development shifts to the efficient production of these fuels. TiH_2_ has been recognized as a potential catalyst for ammonia synthesis.^4^ The subtle differences in the surface structure of the hydride compared to the metal depending on temperature and pressure have been proposed as the origin of this effect.^5^

An alternative solution is the storage of hydrogen in lightweight complex metal hydrides such as NaAlH_4_.^6^ Interestingly, the decomposition/reformation reaction of NaAlH_4_ is only reversible under technically applicable conditions if the material is doped with transition-metal compounds, most efficiently with titanium compounds.^7^ Despite 25 years of investigation, the catalytic mechanism of Ti in NaAlH_4_ remains controversial, primarily due to the complexity of the material.^8^ Under the conditions applied, the Ti is reduced to an oxidation state near zero, and reversible hydrogenation of it (TiH_*x*≃0_ ↔TiH_*x*≤2_) is likely to occur upon hydrogenation cycling.^9,10^ In general, Ti has been found to be a promising additive enhancing kinetics in other metal hydride systems such as MgH_2_.^11^ A strategy toward deeper insight is thus to reduce the complexity of the material systems and study solely the surface properties of Ti–H at relevant conditions.

**Figure 1:**
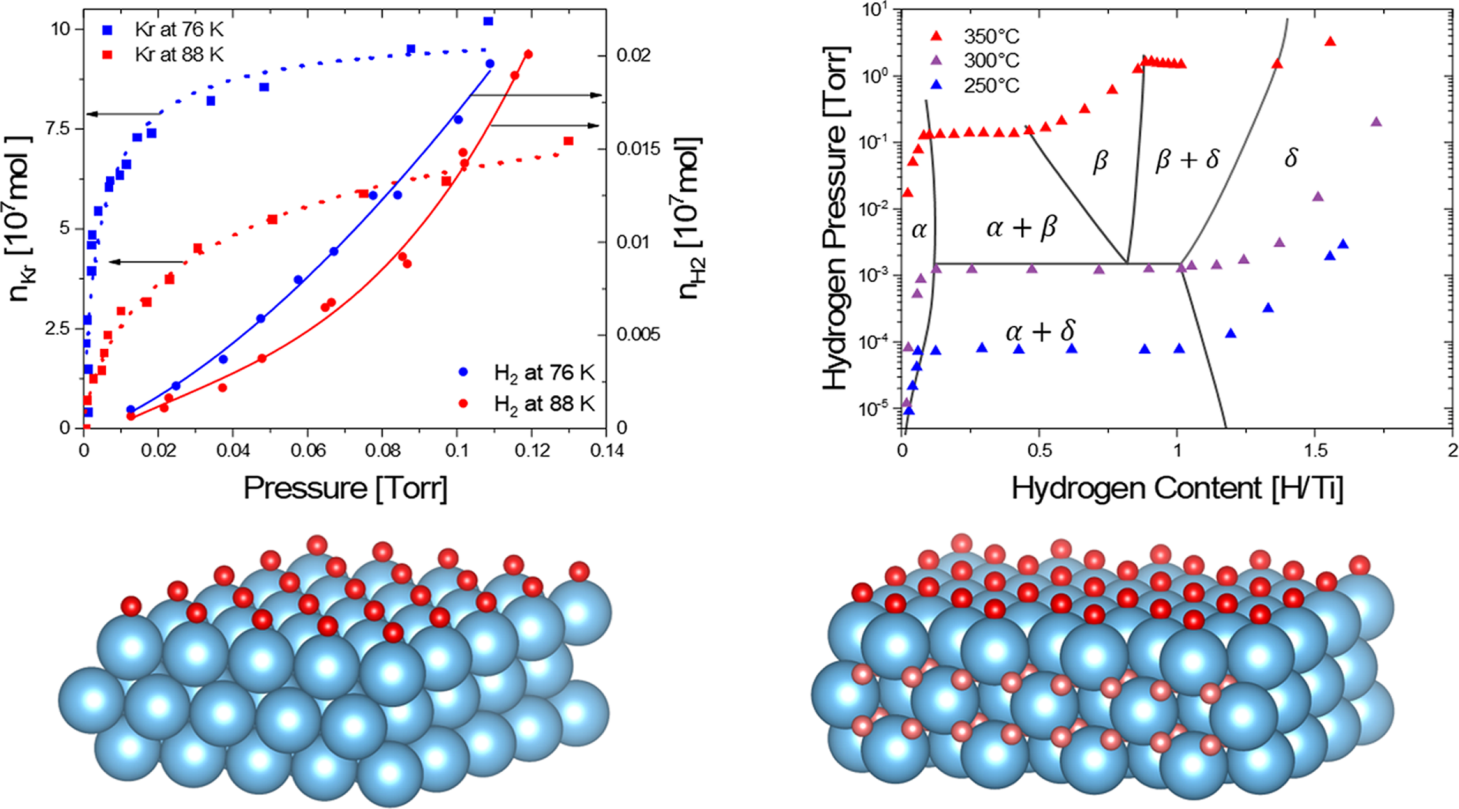
Left panel: adsorption of gases (Kr, H_2_) on Ti (adsorption data from ref (12)). Right panel: hydrogen sorption in Ti (sorption data from ref (13)) as represented by pressure-composition isotherms (pcT). It is common to display the pressure-coverage dependence with *x*-axis as pressure in surface science. The bulk pressure-composition dependence uses the hydrogen content as *x*-axis. The various bulk phases are indicated.^14^ Bottom: Schematic crystal structures of hydrogen adsorbed on hexagonal α-Ti (left) and on cubic δ-TiH_2_ (right) are shown.

Because of its relevance, as well as its apparent simplicity, the interaction of hydrogen with metallic surfaces is generally well studied.^15^ However, knowledge on surface properties is limited when it comes to the class of metals, which absorb large quantities of hydrogen, such as titanium. Their surface properties depend on the state of the underlying bulk, in contrast to nonabsorbing metals, where the surface coverage is solely described by gas–surface interactions.^16^ This complicates the description of the state of matter. Krypton can be considered of not being absorbed by bulk Ti. The coverage of Ti by Kr is thus perfectly described by a Langmuir isotherm (see Figure 1). However, even at liquid nitrogen temperatures, hydrogen adsorption does not follow such a trend. Wilde and Fukutani relate the coverage of hydrogen on Ti to the bulk concentration of hydrogen in Ti.^17^ They explained the time dependence of the surface coverage by hydrogen diffusion into the bulk; it is indeed long known that the surface is not fully hydrogenated until the volume is saturated.^18^ This means that the bulk hydrogen–titanium phase diagram as displayed in Figure 1 predefines the surface properties. An experiment probing the surface of Ti must therefore control the bulk phase as well.

The preparation of clean surfaces of typical hydride-forming metals such as Ti requires ultrahigh vacuum (UHV) conditions due to their reactivity.^19,20^ UHV technology is incompatible with high hydrogen pressure required to form hydrides. Thus, most of the studies rely on postmortem samples, where the samples were prepared elsewhere, and the state of hydrogenation was quenched,^21^ and/or the surface of a stable hydride was (re-) generated by fracturing the sample.^22^ This impedes the measurement of surface properties as a function of bulk hydrogen concentration. In this paper, we demonstrate the membrane approach to determine the surface hydrogen concentration in TiH_*x*_. A hydrogen-permeable Ti-coated Pd membrane enables a simple control of the hydrogen pressure in the UHV chamber. Palladium stands out from the group of H-absorbing metals, partly due to its importance in heterogeneous catalysis. Furthermore, in contrast to all other hydride-forming metals,^19,20^ Pd is practically inert to oxidation^23,24^ and has a high hydrogen permeability,^25^ making it ideal as the material for the feed surface side and bulk of the membrane.^26^ Using electron energy loss spectroscopy (EELS), we are able to determine a full surface and bulk pressure-composition isotherm (pcT). The experiments are supported by state-of-the-art density functional theory (DFT) calculations of the TiH_*x*_ surface. The joint results are discussed in conjunction with the current understanding that hydrogen vacancies on the TiH_2_ surface are the preferred site for nitrogen activation. The implications on the use of titanium as a hydrogen dissociation catalyst promoting hydrogen sorption in magnesium and as a hydrogen gateway for the hydrogen sorption in sodium alanate are also considered.

## 2 Experimental Section

### 2.1 Membrane Holder and Chamber Setup

**Figure 2:**
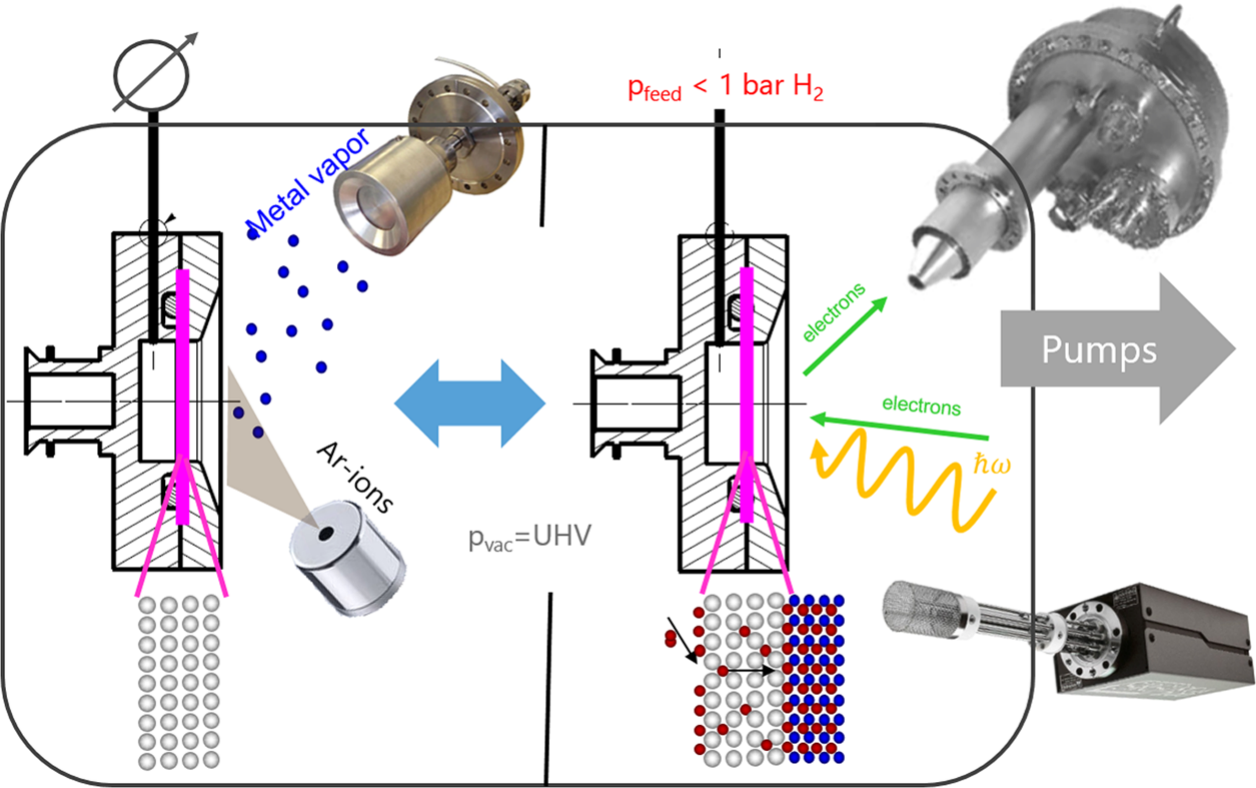
Scheme of the apparatus used to deposit Ti thin films on the Pd membrane (left part) and the analysis by electron spectroscopy (right part). Both parts are integrated into the same vacuum chamber, separated only by metal shielding. The membrane holder is fixed on an *xyz* manipulator moving between preparation and analysis steps. See Experimental Sections 2.1–2.3 for details.

The membrane sample holder is integrated into a cylindrical ultrahigh vacuum chamber with a preparation and an analysis level separated by an aperture (Figure 2). The analysis level consists of a VSW Class100 hemispherical electron analyzer equipped with a single-channel electron multiplier. A dual anode X-ray source (Prevac RS 40B1) is mounted at a 58° angle, and a tuneable electron source (Specs EQ 22/35) is mounted at 50° with respect to the entrance of the electron analyzer. The sample preparation level includes a variable energy argon ion source (Leybold), a magnetron sputter deposition source (AJA A300 XP), and an active capacitance pressure gauge (Pfeiffer CMR 363). Additionally, the chamber is equipped with a quadrupol mass analyzer (SRS RGA 100) and two turbomolecular pumps (Pfeiffer HiPace 300 and HiPace 80).

### 2.2 Titanium Deposition

The palladium membrane was cleaned by argon ion etching at 5 keV until Auger electron spectroscopy (AES) showed only Pd peaks. Titanium thin films were grown on the palladium membrane by RF magnetron sputtering at 80 W for 30 min from a titanium target (99.995%). A film thickness of 37 nm was determined by SEM on FIB cuts from titanium films grown on polished sapphire substrates.

### 2.3 Electron Spectroscopy

**Figure 3:**
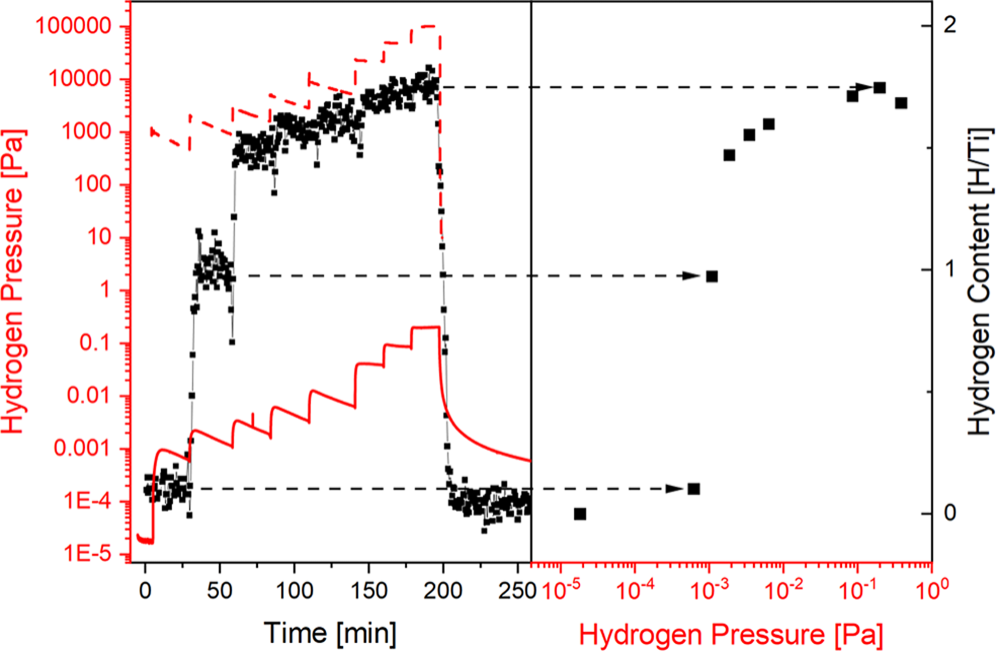
Left: Typical measurement cycle to derive a pressure-composition isotherm as described in Section 2.3, here at 225 °C. Red continuous and dashed lines are the hydrogen pressures at the UHV and feed side of the membrane, respectively. The hydrogen content at the surface is given by black squares. Significant hydrogen uptake does not take place below 10^–3^ Pa. Vice versa, the thin film has desorbed all its hydrogen when the pressure falls below this value, demonstrating the reversibility of the system. Right: Construction of the pressure-composition isotherm by displaying the hydrogen content as a function of the hydrogen pressure in the UHV chamber.

The hydrogenation of titanium was followed operando by electron spectroscopy measurements. At the beginning of each measurement series, the sample was thoroughly characterized by standard X-ray photoelectron spectroscopy (XPS), AES, and EELS measurements. To follow the hydrogenation, a series of spectra was acquired sequentially. The first five were used to characterize the initial state of the system, then the hydrogen pressure was applied, and the recording was stopped once the new equilibrium state was reached (see Figure 3). This procedure was repeated for all subsequent pressure steps. At low hydrogen pressure, the feeding system was operated as a closed system, leading to a small pressure drop during the measurement. At higher pressures, the applied hydrogen pressure was actively regulated by the pressure reducer leading to the flat plateaus during the measurement cycle. At the maximum hydrogen pressure of approximately 1 bar, the sample was characterized by XPS, AES, and EELS again. The hydrogen desorption was measured by a series of sequential spectra, while the hydrogen supply was pumped by a turbomolecular pump. Detailed acquisition parameters of all spectra shown are given in Table 1.

**Table 1:** Acquisition Parameters for the Electron Spectroscopy Measurements Shown

spectrum	*h*ν/*E*_Kin_ [eV]	*U* [kV]	*I* [mA]	range (*E*_Kin_) [eV]	Δ*E* [meV]	pass energy [eV]	acquisition time/point [s]
XPS survey	1486	15	26.6	200–1500	500	100	0.4
XPS Ti 2p	1486	15	26.6	980–1050	100	30	4.8
XPS VB	1486	15	26.6	1465–1490	100	100	16
EELS	2000	2	1 μA	1975–2008	200	30	0.2

### 2.4 Data Treatment

**Figure 4:**
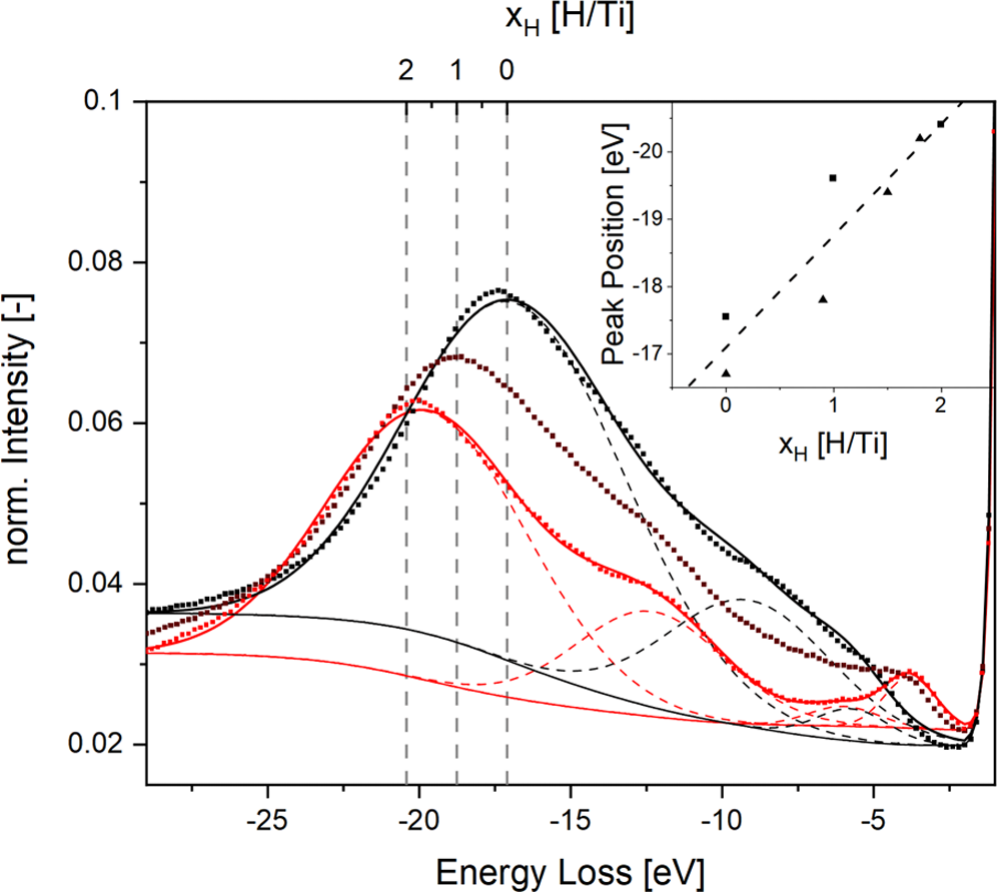
Electron energy loss spectra of titanium at 225 °C exposed to 4.6, 9.6, and 1009.4 mbar hydrogen back pressure, respectively (black, claret, and red). The spectra show features from plasmon excitation around 18–20 eV and interband transitions at lower energies, respectively. The inset shows the calibration derived from a linear relationship between the hydrogen concentration and the plasmon loss peak based on literature data.^38,39^

All data treatment was performed using Rstudio^27,28^ and CasaXPS (version 2.3.22). The elastic recoil peak of EELS spectra was fitted with a Gaussian function that was shifted to 0 eV energy loss. The loss spectrum was fitted with a Shirley background and four Gaussian functions (see Figure 4) to extract peak positions of different plasmon features. The pressure-composition isotherm points are calculated from averaged peak positions of all of the spectra once equilibrium is reached. The Ti 2p XPS spectrum was fitted using a Shirley background and the asymmetric LA(1.2,2,7) lineshape in CasaXPS. The Ti 2p_3/2_ peak was shifted to 453.7 eV for binding energy referencing.^29^

### 2.5 DFT Calculations

All calculations were performed within density functional theory (DFT) with periodic plane-wave basis set as implemented in Vienna *Ab initio* Simulation Package.^30,31^ The calculations parameters were as follows: cutoff energy for the basis set expansion 500 eV; the *k*-point sampling density *k*·*a* ≥ 50 for TiH_2_ and *k*·*a* ≥ 100 for Ti; the convergence criteria for electronic degrees of freedom was 10^–6^ eV/Å; for the structural relaxations, the conjugated gradient method with convergence 10^–2^ eV/Å was used; projected augmented wave potentials (PAW)^32,33^ for atoms with electronic configuration 3p^6^3d^2^4s^2^ for Ti and 1s^1^ for H; and Perdew, Burke, Ernzerhof (PBE) exchange-correlation functional.^34^ The surface calculations were performed in the slab geometry with a minimum of 15 Å, of vacuum separating slab periodic images. For the (111) surface of TiH_2_, five atomic layers were used with the 2 × 2 *R*45 surface. The symmetric slab was relaxed with respect to atomic positions, and later the bottom two atomic layers were frozen. Additional calculations were performed for (100) and (110) facets to obtain the work function. For Ti(0001), (101̅1), and (112̅0), slabs were constructed to calculate the work function. Hydrogen diffusion paths were calculated with the nudged elastic band method (NEB).^35^ Vacancy formation was calculated by removing H atoms from the bulk, surface, or subsurface layers in TiH_2_; for the bulk, the 2 × 2 × 2 supercell was used.

## 3 Results

As outlined in the Section 1, the surface of TiH_*x*_ is the origin of its peculiar catalytic properties. Its characterization requires a method being able to quantify the surface hydrogen content at catalytically relevant pressures and temperature, which we describe in Section 3.1. We apply the method to determine equilibrium pressure-composition isotherms in Section 3.2. Properties extracted from the pcT are also calculated by DFT methods presented in Section 3.3.

### 3.1 Method to Determine Surface Hydrogen Content by Reflecting Electron Energy Loss Spectroscopy (REELS)

**Figure 5:**
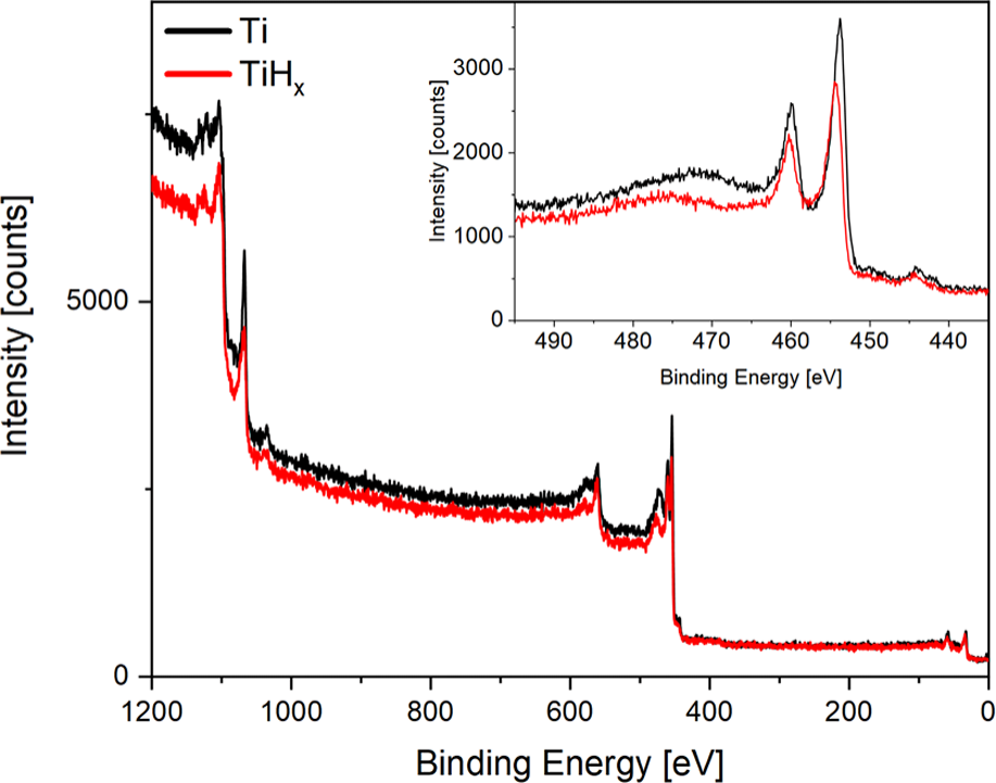
Survey X-ray photoelectron spectra of freshly prepared titanium (black) and TiH_*x*_ (red). No surface contamination such as carbon (*E*_B_ ≃ 286 eV) and oxygen (*E*_B_ ≃ 530 eV) is observed during the hydrogenation experiments. The inset shows the narrow-scan spectra of the Ti 2p region, where the Ti 2p 3/2 peak shifts by 0.59 eV upon hydrogenation, in good agreement with literature.^38^

The experimental setup enables the reproducible deposition of Ti onto a Pd membrane, which links the chemical potential of the thin film in UHV with the gas applied on the feed side (Figure 2).^26^ The exact pressure on the vacuum side depends on the specific material parameters and is thus measured with a quadrupole mass spectrometer installed near to the membrane holder. In addition to the control of the pressure, the palladium membrane delivers atomic hydrogen, practically without impurities, to the thin film and its surface.^36,37^ To probe this surface, we are in need of a surface-sensitive method to quantify the hydrogen content as a function of pressure. Since the H 1s electrons are involved in chemical binding and thus are not true core levels, photoemission spectroscopy is inappropriate for hydrogen quantification, while it is used for all other elements (see Figure 5).

Recently, the quantification of hydrogen by analysis of plasmon excitations has been established in some hydrogen metal systems.^40,41^ The corresponding measurements, so-called electron energy loss spectroscopy, are relatively simple and can be integrated both in bulk (e.g., transmission electron microscopy) and surface characterization techniques such as utilized here (reflecting electron energy loss, REELS).^42^ The mean free path length of REELS using 2000 eV electrons is around 3 nm.^43^ The information depth can be varied using different excitation energies. The corresponding results will be presented in the future.

A REELS spectrum shows features from collective as well individual particle interactions, i.e., the plasmon peak as well as interband transitions (Figure 4).^43^ In the ideal free-electron model, the plasmon energy depends solely on the number of free electrons. The nonideal behavior of the electrons can be included in the effective electron mass *m* and the dielectric constant ϵ.^44^ Hydrogen intercalation will affect all three parameters.^45^ In this paper, we can omit ab initio calculations of the EELS spectrum of the hydrogen–titanium system, as some specific compositions were already measured in the past.^38,39^ From these reference measurements, we derive a calibration curve to quantify the hydrogen content *x*_H_ by relating it to the operando measured plasmon peak energy ω_p_ = *a* + *b*·*x*_H_, with *a* = −17.10 ± 0.38 and *b* = 1.66 ± 0.30 (see Figure 4).

Subsequently after the quantification of the hydrogen content, X-ray photoelectron spectroscopy (XPS) and/or Auger electron spectroscopy (AES) are utilized to determine all other elements present. The system was designed to minimize surface contamination, and indeed within a typical measurement lasting a few hours, no contamination is detectable (Figure 5). After a hydrogenation cycle, the Ti layer was removed by Ar sputtering. For a new measurement, Ti was freshly deposited and hydrogenated.

### 3.2 Surface Pressure-Composition Isotherms by REELS

**Figure 6:**
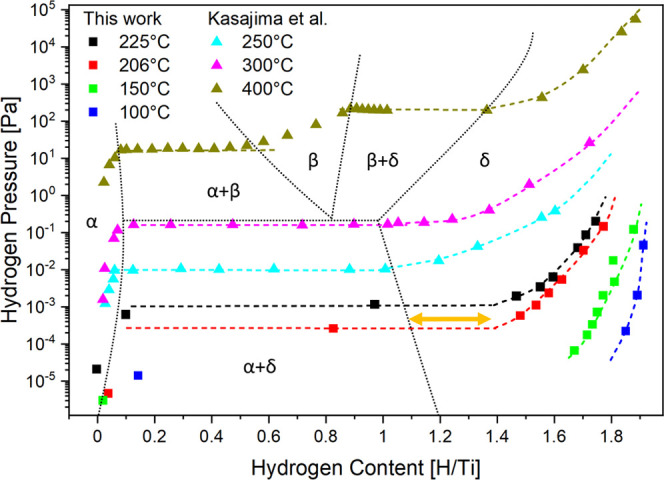
Pressure-composition isotherms of the titanium–hydrogen system measured by electron energy loss spectroscopy (this work) and literature data measured by the Sieverts method from Kasajima et al.^13^ The bulk titanium–hydrogen phase diagram is indicated with dotted lines and labels of the different phases. The different plateau length of the bulk and surface two-phase regime is indicated by an orange arrow.

For hydrogenation, the feed side of the membrane is exposed to hydrogen, which diffuses through the Pd membrane and subsequently through the Ti layer to its surface. The corresponding chemical potential inside the membrane decreases from the high-pressure side, where it is linked to the feed pressure, to the UHV side (see Figure 3), where it is linked to the UHV pressure building up upon hydrogenation. Assuming that both Pd and Ti have practically no dissociation/recombination barrier for hydrogen (see Figure 8), the surface pressure-composition isotherms can be extracted from equilibrium (steady state) values of surface hydrogen concentration and UHV pressure. For an isotherm, the feed pressure is step wise increased, and the hydrogen pressure near the membrane and the hydrogen content are continuously measured. After reaching a quasiequilibrium, the pressure and concentration values are plotted in a pcT diagram (Figures 3 and 6).

Below 100 °C, the method is impeded by the very slow kinetics and the extremely low-plateau pressures at these temperatures. Most complete pressure-composition isotherms were thus obtained for a temperature around 200 °C (Figure 6). Unfortunately, measurements at higher temperatures are impeded by the alloying of Ti into the underlying Pd membrane changing the membrane irreversibly, as can be observed using XPS.

**Figure 7:**
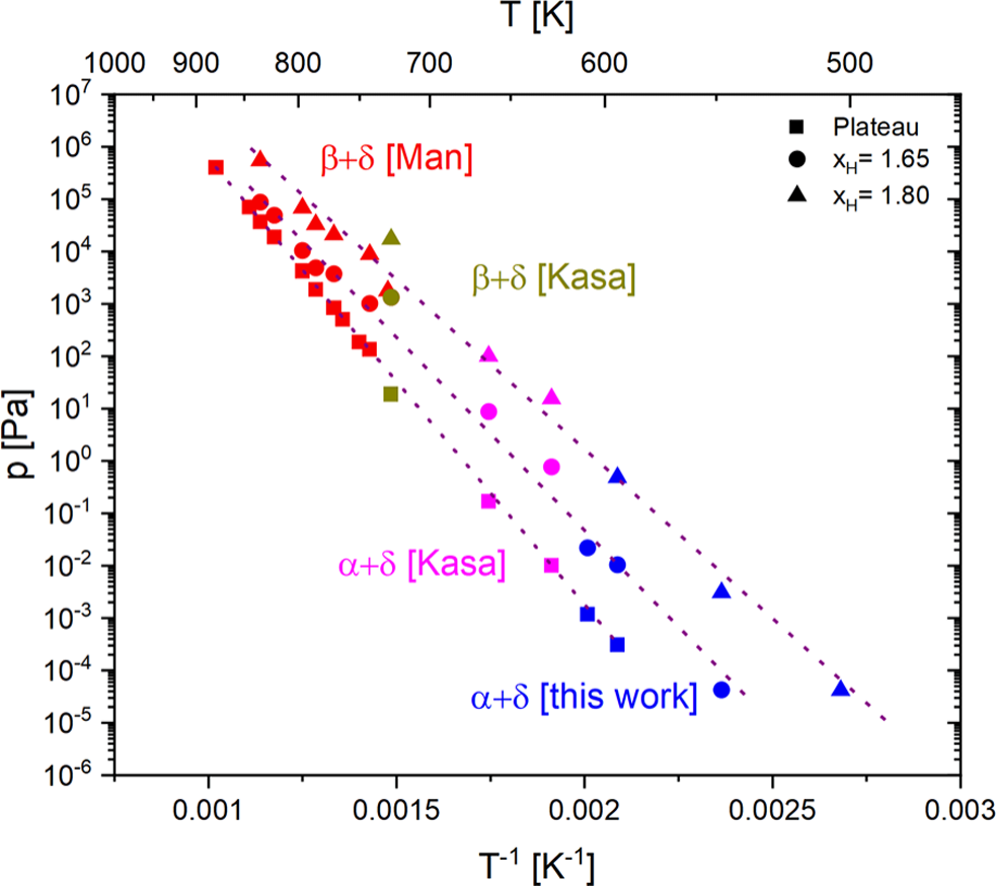
Van’t Hoff analysis of the hydrogen pressure in the plateaus (squares) and at two concentrations *x*_H_ = 1.65 (spheres) and *x*_H_ = 1.8 (triangles). The heat of formation (Δ*H*_δ_) decreases with the increasing hydrogen content. A good agreement between bulk literature data (Kasajima^13^ and Manchester^14^) and our surface measurements over 9 orders of magnitude is observed.

We determine the phase from the pcT diagram since the EELS measurement is not directly sensitive to the crystal structure. The measurements shown in Figure 6 resolve the δ-TiH_*x*≤2_ phase in great detail and are fitted by a model from Wang.^46^ The pcT shows only one plateau, as expected at these temperatures, i.e., the hydrogenation proceeds from the α-phase directly to the δ-phase. This is also in agreement with the EELS data (Figure 4), showing continuous growth/decline of features without the appearance of intermediate structures. The temperature dependence of plateau pressures (Figure 7) matches an extrapolation of literature data over 9 orders of magnitude, i.e., surface and bulk plateaus of the Ti to TiH_2_ phase transformation have practically the same thermodynamic properties (Δ*H*_α__→δ_, Δ*S*_α__→δ_). The heat of formation decreases with increasing hydrogen content from Δ*H*_α→δ_ = −141 kJ(mol H_2_)^−1^ in the plateau to Δ*H*_δ_ = −107 kJ(mol H_2_)^−1^ at *x*_H_ = 1.8. While the plateau pressures are equal, the plateau is longer on the TiH_*x*_ surface, as indicated by the orange arrow in Figure 6. The implications of these observations are discussed in Section 4.2.

### 3.3 DFT Calculations

Modeling the experimental isotherms is based on the assumption of specific models, resulting in an uncertainty of the derived parameters such as the heats of formation. These parameters can also be assessed by electronic structure calculations. Details of the DFT calculations are given in the Experimental Section 2.5. Calculated formation energies for hydrogen vacancies and the charge on H in TiH_2_ are shown in Table 2.

**Table 2:** Formation Energy of H Vacancies *V*_H_^*i*^ and the Charge *q*_H_^*i*^ in TiH_2_ at the (111) Surface, First Subsurface, Second Subsurface, and in the Bulk^a^

	surface	first subsurface	second subsurface	bulk
TiH_2_: *V*_H_ (eV/H)	1.46	0.87	0.72	0.72
TiH_2_: *q*_H_ (e/H)	–0.65	–0.7	–0.69	–0.67
1ML H@Ti (0001): *V*_H_ (eV/H)	1.14			
1ML H@Ti (0001): *q*_H_ (e/H)	–0.66			

a The energies are with respect to the H_2_ molecule in the gas phase.

**Figure 8:**
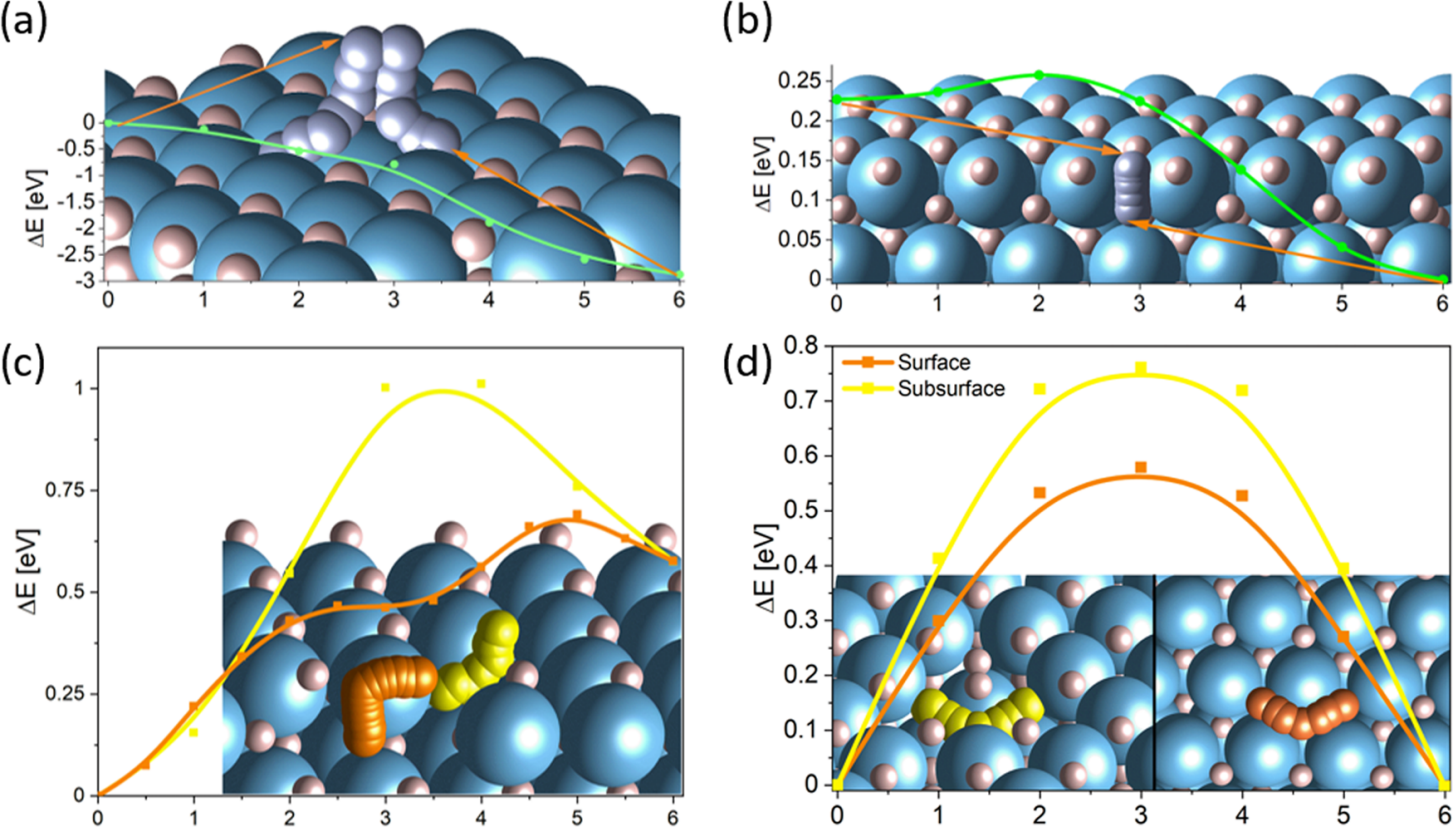
Visualizations of various transition-state NEB calculations. Titanium atoms are blue, hydrogen is light pink, diffusing hydrogen atoms are gray, and hydrogen vacancies are yellow or orange. (a) Absorption of a dihydrogen molecule shows no activation barrier on a TiH_2_(111) surface. (b) Diffusion of a hydrogen atom from the TiH_2_(111) surface to the subsurface is favored by 0.22 eV and inhibited by a very small barrier of 0.03 eV. (c) Diffusion of a hydrogen vacancy along the surface followed by a jump to the subsurface (orange) is associated with a barrier of 0.12 eV, while the jump to the subsurface followed by lateral diffusion (yellow) exhibits a barrier of 0.48 eV. (d) Lateral diffusion of a hydrogen vacancy along the surface (orange) and subsurface (yellow) is associated with barriers of 0.5 and 0.75 eV, respectively.

We assume an equilibrium state between the titanium hydride surface and the hydrogen pressure inside the UHV chamber for the construction of pcT curves. To strengthen this argument, we calculated the transition states of hydrogen dissociating on a TiH_2_ surface and diffusion of hydrogen in/on the titanium surface (Figure 8). The absorption of a dihydrogen molecule on the TiH_2_(111) surface is favored by 2.92 eV and encounters no activation barrier (Figure 8a). The diffusion of a hydrogen atom from the surface to a subsurface vacancy is also energetically favored by 0.22 eV and encounters an activation barrier of 0.03 eV that is negligible under the experimental conditions (Figure 8b). These calculations support our assumptions for the construction of pcT curves.

Various pathways are possible for the diffusion of a hydrogen atom or hydrogen vacancy in TiH_2_. A complete compilation of all possible pathways is beyond the scope of this paper; therefore, we calculated some extreme cases giving qualitative trends for the mobility of hydrogen in TiH_2_, e.g., two calculated diffusion pathways indicate that vacancy hopping from the surface to the subsurface lattice site below followed by diffusion on the subsurface has an energy barrier of 0.48 eV, while diffusion of the vacancy on the surface layer followed by a vertical jump to the subsurface is energetically preferred with the barrier of 0.12 eV (Figure 8c). The vacancy is more stable at the first subsurface layer by 0.58 eV. H vacancy diffusion in a plane parallel to the surface is related to a barrier 0.5 or 0.75 eV for the surface and subsurface planes, respectively (Figure 8d).

We calculated the adsorption energies of hydrogen on Ti and TiH_2_ surfaces to construct a potential-energy surface (Figure 10). The most favorable site is the TiH_2_ surface (see also Table 2). Hydrogen adsorption becomes more favorable with increasing coverage on the Ti(0001) surface and vice versa for the TiH_2_(111) surface.

An additional surface parameter, which can be compared to the experiment is the work function. Experimentally, we find no work function shift within the experimental uncertainty of 0.1 eV. The chemical shift of the Ti 2p_3/2_ core level is +0.59 eV upon hydrogenation (see Figure 5), equal to the Auger parameter difference Δα′ = *E*_K_(TiL_3_M_2,3_M_4,5_) + *E*_B_(Ti 2p_3/2_) = 0.65 eV. This shows that the observed shifts are purely due to changes in the titanium environment.^47,48^ Calculated work functions are 4.4 eV for (111), 3.1 eV for (110), and 4.5 eV for (100) facets of TiH_2_; for Ti, they are 4.2, 3.9, and 3.2 eV for (0001), (101̅1), and (112̅0) facets, respectively. Experiment and theory are in good agreement since we are working with polycrystalline surfaces.

### 3.4 Permeation Kinetics

**Figure 9:**
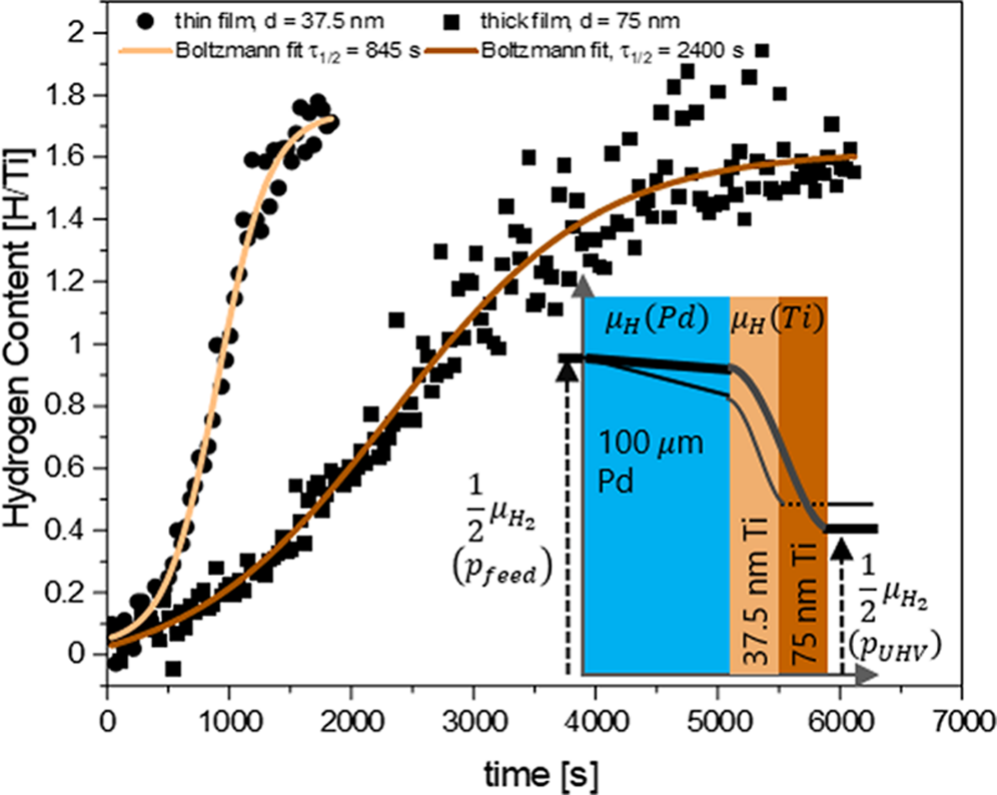
Time evolution of the surface concentration of TiH_*x*_ thin films with two thicknesses applied to the same feed pressure *p*_feed_ = 960 mbar at *T* = 100 °C. The inset is a sketch illustrating the local chemical potential gradient of hydrogen diffusing through a Pd membrane coated with a Ti film.

Figure 9 shows the development of the surface hydrogen concentration on Ti thin films with two different thicknesses *l*_Ti_. The measured half time of the thick film is 4 times longer than the twice thinner one as expected by the simple model of hydrogen diffusing into a plane^49^
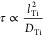
1where *D*_Ti_ is the diffusion coefficient in the Ti layer. However, the time constant includes technical parameters such as the pumping speed of the system and is thus not easily converted to fundamental parameters. The measurements evidence that after a transient response, the hydrogen concentration reaches a steady state, which links surface concentration and UHV pressure.

## 4 Discussion

### 4.1 Membrane Method

The investigation of the dynamic surface properties of TiH_2_ presented in this paper is a proof of concept of the membrane method for the investigation of the surface of metal hydrides. The “underlying” idea, the hydrogenation of a metal layer in UHV by depositing it on a hydride substrate, usually PdH_*x*_, has been developed by Krozer et al. at the end of the last century.^36^ In this paper, we extend the application to the operando measurement of surface hydrogen pressure-composition isotherms. Furthermore, it opens the real-time observation of hydrogen permeation through membranes. The reachable pressure depends on the material to be studied: a material such as Ti without a dissociation barrier for hydrogen is in quasiequilibrium with the surrounding gas, which limits the pressure to the maximum pressure of the vacuum system and its compartments (see Section 3.1). This limit is a disadvantage. However, at the same time, the straightforward outcome of a measurement is whether the material exhibits a significant dissociation barrier. These measurements can be performed practically without contamination (see Figure 5), being a crucial prerequisite of studying most hydride-forming metals and metal alloys. Materials such as Mg exhibiting a high dissociation barrier are in quasiequilibrium with the feed pressure allowing higher pressure ranges.^26^ Furthermore, access to various surface characterization methods and manipulation of the material by sputtering or gas dosing facilitates well-controlled studies, such as the impact of oxygen contamination or the hydrogenation of layered materials. As a proof of concept, we determined fundamental parameters such as the density of electron states in TiH_*x*_ (Figure 11) and chemical shift originating from hydrogenation. It is worth mentioning that the inelastic loss features in XPS (e.g., Figure 5) and Auger electron spectroscopy (not shown) reflect the REELS spectra used to determine the hydrogen concentration. This means that also XPS core-level spectroscopy can be used for quantification by including the loss features despite the relatively small chemical shifts of core levels (≃0.5 eV). The plasmon peak shift has been observed in a variety of materials such as light metal hydrides,^45^ yttrium hydride,^50^ and niobium hydride.^51^

### 4.2 Surface and Bulk Hydrogen in Titanium

At temperatures below 573 K, titanium and hydrogen form solid solutions (α–Ti) as well as TiH_2_ (δ−Ti).^14^ Both phases are very stable (Δ*H*_sol_ ≃ −0.4 eV/H and Δ*H*_hyd_ ≃ −0.8 eV/H, respectively). At higher temperatures, an additional phase is formed (β-Ti, corresponding to TiH_1_, see also Figure 1). The equilibrium pressures of the α–δ plateau are in the low UHV range at room temperature impeding their correct determination and are further impeded by the slow kinetics due to relatively high surface contamination in bulk experiments. Published data on bulk samples start at 523 K (Figure 6). Being compatible with UHV conditions, we were able to identify a plateau already at 479 K, and its full length at 498 K. With our REELS method, we probe the surface and subsurface region (for simplicity called near surface). In the van’t Hoff plot, the corresponding near-surface plateau pressures lie on the function extrapolated from the data points measured in bulk samples, i.e., the heat of absorption (Δ*H*_α_–δ) is similar in the bulk and the near surface. The relative stability of the phases is given by the plateau length, which is significantly larger in our surface-sensitive measurements. We can conclude that the relative heat of absorption of the surface hydride phase (Δ*H*_δ_^*^/Δ*H*_α_^*^) is larger compared to the bulk phase. This finding is supported by calculated vacancy formation energies of *V*_H_ = 1.46 eV/H (140.87 kJ/H) at the TiH_2_ surface and *V*_H_ = 0.72 eV/H (69.47 kJ/H) in TiH_2_ bulk (see Table 2).

This finding only applies to the plateau region, where the hydrogen content in the two phases is invariant and only the relative amount of the phases changes. The heat of absorption is strongly dependent on the hydrogen concentration in the δ-TiH phase.^52^ We resolve this phase very well and can model this dependence. We find −141 kJ (mol H_2_)^−1^ at *x*_H_ = 1.41 and −122 kJ (mol H_2_)^−1^ at *x*_H_ = 1.65. Arita et al. report −148 kJ (mol H_2_)^−1^ + 2.7·*x*_H_ kJ (mol H_2_)^−1^ and Dantzer gives Δ*H*_δ_ (*x*_H_ = 1.41) = −157 kJ (mol H_2_)^−1^ to −125.5 kJ (mol H_2_)^−1^ at *x*_H_ = 1.85.

**Figure 10:**
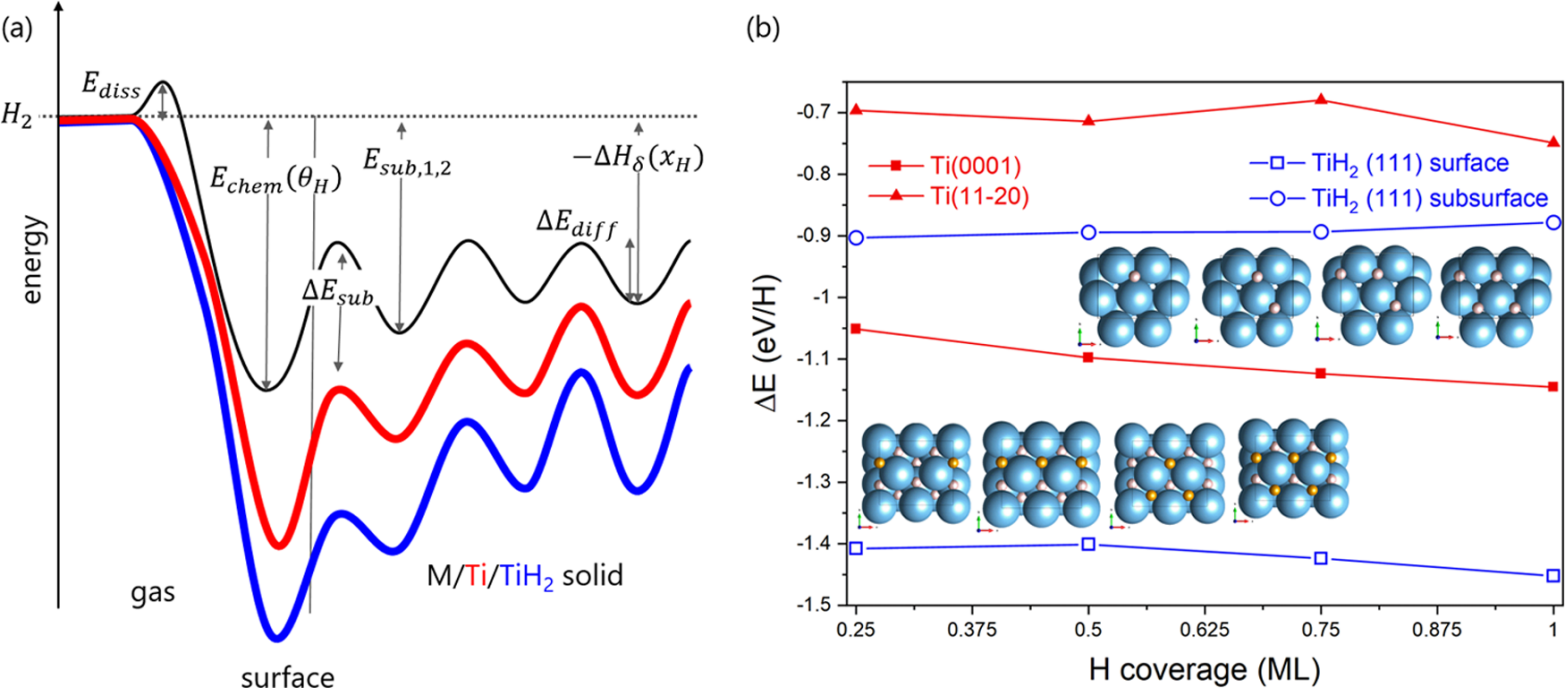
(a) Simplified one-dimensional potential-energy surface (PES) of hydrogen on/in Ti and TiH_2_ are in red and blue, respectively. The black curve represents the PES of an archetypal d-metal such as Ni. (b) Energy of hydrogen adsorption on two titanium surfaces (red) and on the TiH_2_ surface and the subsurface (blue) as a function of hydrogen coverage.

To facilitate the discussion, a simplified potential energy surface (PES) is sketched in Figure 10. Most relevant energies determining the thermostatic and kinetic properties of the hydrogen solid interaction are indicated. The black curve is the archetypal PES of hydrogen on/in d-metals such as Ni exhibiting a dissociation barrier, a strong chemisorption, and a less strongly bound hydrogen absorption on surface and subsurface sites.^53−56^ Eventually, energies approach the values of the heat of absorption of hydrogen in the solid solution and heat of hydride formation, respectively. The calculations show a much stronger binding of hydrogen on the surface than in the subsurface and bulk expressed by the formation of a hydrogen vacancy in TiH_2_. Our experimental values agree with the energy of chemisorption *E*_chem_ on Ti to vary between 0.76 and 0.43 eV/H for low and high coverage, respectively.^57,58^ It is worth noting that these values are smaller than the bulk heats of solution/formation and much smaller than the calculated value (Table 2). This apparent disagreement with the experimental near-surface pcT measurements (Figure 6) is explained as follows: REELS probes several monolayers deep, i.e., it is to be compared to an average of the surface and bulk values in Table 2. With the very high formation energy of 1.4 eV/H at the surface, which corresponds to hydrogen pressures below the experimental limit, dynamic exchange of hydrogen is assumed to take place in the subsurface layers only. With this, the agreement between the experiment and theory is excellent: the measured “near-surface” enthalpy of formation is 0.82 eV/H (see the discussion above and refs (52, 59)). Wilde and Fukutani^17^ estimated the enthalpy of chemisorption to 0.92 eV/H based on dynamic experiments. The calculated subsurface and bulk enthalpies are 0.87 and 0.72 eV/H, respectively (Table 2). The averaging of surface and bulk in our measurements also explains the relative difference in plateau length being too small compared to the vacancy formation energies. A true surface pcT should exhibit a much wider plateau and conversely a very narrow substoichiometric TiH_2_ phase. Furthermore, the surface hydride does not exist without the underlying bulk hydride. This explains why the overall equilibrium pressures measured at the surface and bulk hydride do not differ: the bulk “pins” the surface. On the other hand, if the influence of the surface is significant, the solubility of hydrogen depends on the macroscopic shape of the sample, as has been found in the related material system Nb-H.^60^ The coexistence lines in Figure 6 are estimations, and the corresponding discussion is a simplification of reality. A more detailed theoretical framework is given in ref (61), which also corroborates our findings.

The explanations above rely on the implicit assumption that the barriers for dissociation and hopping between surface and subsurface sites are negligible, which is in good agreement with literature on Ti single crystals.^17^ The experiments confirm the assumption of generally very small barriers between gas-phase hydrogen and bulk hydrogen. Further details are gained from density functional theory calculation of the transition states of hydrogen on various pathways on/in Ti and TiH_2_. Figure 8 is a compilation of the various calculations. Summarizing, the calculations corroborate that Ti and TiH_2_ do not exhibit a dissociation barrier (Figure 8a). The heats of chemisorption *E*_chem_ on Ti, though, are smaller than on TiH_2_ and depend on the crystal facet (Figure 10b). Furthermore, *E*_chem_ (H on Ti) increases with coverage, being the precursor of the formation of TiH_2_. Vice versa, the energy of the understoichiometric TiH_2_ is slightly larger than for unity coverage, pinpointing to the existence of vacancies at the surface. The mobility of hydrogen toward the bulk depends on the pathways. The hopping of interstitial H from the surface to a subsurface vacancy is thermodynamically advantageous by 0.22 eV and associated with a negligible barrier of 0.03 eV (Figure 8b). Further calculations may be summarized by estimating an averaged energy barrier of 0.5 and 0.75 eV at the surface and in bulk, respectively. This compares well with the experimental values of the bulk hydrogen diffusion in TiH_2_: Bustard et al. report energy barriers of 0.53 eV in TiH_≃1.7_.^62^ This corroborates the relatively high mobility of hydrogen in Ti; the numbers may be compared to hydrogen diffusion in V (Δ*E*_diff_ = 0.045 eV),^63^ Ni (Δ*E*_diff_ = 0.40 eV),^63^ and in Mg (Δ*E*_diff_ = 0.24 eV)^64^ and MgH_2_ (Δ*E*_diff_ ≃1 eV).^65^ The case of Mg is particularly illustrative, as hydrogen diffusion in Mg is relatively fast, while long-range diffusion in MgH_2_ can be considered as nonexistent.

This picture presented above explains not only the experimental behavior of hydrogenation of the Ti film as utilized in this work but also the good getter performance of titanium films. A good getter material has no dissociation barrier, and no surface hydride is formed until the total amount of absorbed hydrogen exceeds the α phase saturation limit, which is indeed observed (e.g., this work, Figure 3, ref (18) and references therein). The energy of adsorption on TiH_2_ is larger than that of H on Ti, but this requires the existence of underlying TiH_2_. Entropy and the fast diffusion of H in bulk drive the hydrogen into the bulk. This makes Ti and related materials good getter materials.^66^ Oxygen or other contaminants will form an oxide layer and are thus detrimental to ad- and further absorption (Figure 10).^67^ In getter pumps, this challenge is overcome pragmatically by regularly depositing a fresh and thus clean Ti layer.

### 4.3 Catalysis

The results presented above confirm some of the assumptions made in the theoretical investigation on the catalytic activity of TiH_2_ for ammonia synthesis by Tsuji et al.^5^ The observed substoichiometry (Figure 6) translates into surface vacancies with high density and high reactivity for N_2_ dissociation and N–H bond formation. The possibility to form H or N vacancies seems to be of general relevance for ammonia synthesis on similar systems, e.g., Ni on LaN.^68^ We also observe a high mobility of hydrogen in TiH_*x*_ that is necessary for a highly active catalyst (Figure 8).

**Figure 11:**
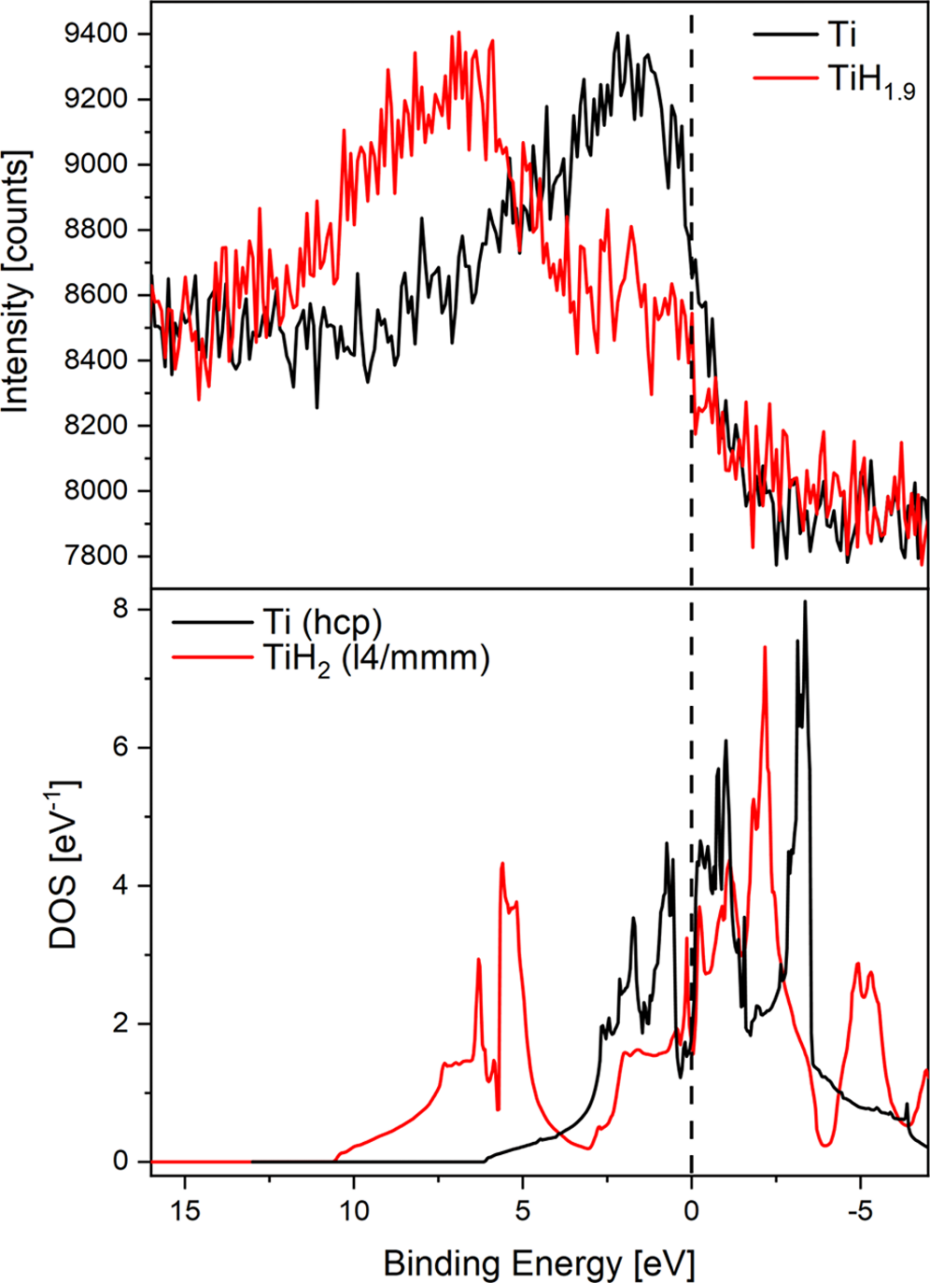
Top graph: valence band X-ray photoelectron spectra of freshly prepared titanium (black) and TiH_1.9_ (red) excited by Al K-α radiation. Bottom graph: calculated total density of the electron states of Ti and TiH_2_.

In our experiments, we observe no work function changes from Ti to TiH_2_. This is in contrast to literature data^67,69^ but in agreement with our DFT calculations and measurements on the polycrystalline surface with various surface planes. The generally low work function of Ti and TiH_2_ is beneficial for the nitrogen activation process by transferring electrons into the 2π* states of N_2_.^5^ Tsuji et al. show that the change in electronic structure is responsible for the N–H bond formation that enables the catalysis on TiH_2_. We measured the electronic structure (Figure 11) and found a very good agreement with the calculated DOS and literature data.^22^ In a simple model, we explain the striking difference between Ti and TiH_2_ by the fact that on TiH_2_ most of the valence electrons are bound more strongly due to their interaction with hydrogen. This seems to inhibit complete nitride formation that results in a loss of catalytic activity, presumably due to the low number of valence electrons available.^70^

### 4.4 Hydrogen Storage

TiH_2_ has empirically been found to be a good hydrogenation catalyst for MgH_2_.^11^ As Mg and Ti are immiscible, it may serve as the archetypal model system of a catalyst particle attached to a metal hydride. The role of TiH_*x*_ as a hydrogenation catalyst may then be that of a hydrogen gateway providing atomic hydrogen to the metal and vice versa. As the typical temperature for hydrogen sorption, Mg–MgH_2_ is around 573 K (300 °C) and operating pressures for application should be above 1 bar; cycling in Ti–TiH_2_ can solely take place in the hydride phase (compare Figure 6). We show here that the corresponding hydrogen mobility is very high, although exclusively possible via vacancies. There is no dissociation barrier on TiH_*x*_ as evidenced by the experiment as well as the theory, in contrast to Mg.^11,71^

The role of TiH_*x*_ as a hydrogenation catalyst in Al + NaH to form NaAlH_4_ is less clear. In the past, the following counterargument against the gateway explanation was given: many catalysts materials such as Pd dissociate hydrogen but are not effective.^8^ However, in contrast to Ni, Pd, Pt, and similar elements, TiH_*x*_ delivers partially negative polarized H^δ−^ (Table 2), which is also the state of hydrogen in NaH and NaAlH_4_.^72,73^ Typical temperatures used for hydrogen cycling in NaAlH_4_ are between 100 and 180 °C, and at pressures up to 10^7^ Pa.^7,74^ Hydrogen mobility in Ti is then again exclusively possible via vacancies (Figure 6). However, in contrast to the simple Ti-Mg system discussed above, the state of Ti in Al–NaH–NaAlH_4_ is less defined.^8^ Particularly, the formation of TiAl_3_ intermetallics has been observed.^10,74,75^ There is some empirical evidence that TiH_2_ dopants are effective catalysts,^76^ which is in line with the above-given explanation. The controversy can be solved by assuming that only a minor amount of Ti is present as TiH_2_ with the majority of Ti alloyed with Al, which results in an averaged formal oxidation number of around one as found by X-ray absorption spectroscopy (XAS/EXAFS).^10,77^ With TiH_2−δ_ always staying in the hydride phase, there is no change in oxidation state expected as indeed found by XAS/EXAFS.^10,77^

In general, the principle of our measurements (a catalytic layer on a metal hydride) mimics the reality of hydrogen sorption in metal hydrides. By starting from very clean surfaces and dosing gases into the UHV system, the impact of contaminations can be studied. Experiments with metal alloy compositions as used in hydrogen storage such as the abovementioned Ti–Al alloy are planned and will give further mechanistic insights.

## 5 Conclusions

The membrane method employed in this study allows for the surface characterization of the contamination-free surfaces of highly reactive materials as titanium using surface science methods. With this, surface pressure-composition isotherms of the titanium–hydrogen system have been measured at low temperatures by reflecting electron energy loss spectroscopy (REELS). Modeling of these pcT curves supported by DFT calculations yields the thermodynamic properties of the surface. They are in excellent agreement with literature data, and the use of UHV techniques extends the parameter space to lower temperatures and pressures. The equilibrium pressure is defined by the bulk properties, although the surface hydride is more stable, as confirmed by DFT calculations. The wide stability range of the substoichiometric (or defective) TiH_2_ surface is discussed in view of these defects being active sites for ammonia synthesis and for the hydrogen sorption in magnesium and alanates.

## References

[ref1] Schlapbach L.; Züttel A. Hydrogen-storage materials for mobile applications. Nature 2001, 414, 353–358. 10.1038/35104634. 11713542

[ref2] Felderhoff M.; Weidenthaler C.; von Helmolt R.; Eberle U. Hydrogen storage: the remaining scientific and technological challenges. Phys. Chem. Chem. Phys. 2007, 9, 2643–2653. 10.1039/b701563c. 17627309

[ref3] Aguey-Zinsou F.; Modi P. Room temperature metal hydrides for stationary and heat storage applications: A review. Front. Energy Res. 2021, 9, 616115 10.3389/fenrg.2021.616115.

[ref4] Kobayashi Y.; Tang Y.; Kageyama T.; Yamashita H.; Masuda N.; Hosokawa S.; Kageyama H. Titanium-based hydrides as heterogeneous catalysts for ammonia synthesis. J. Am. Chem. Soc. 2017, 139, 18240–18246. 10.1021/jacs.7b08891. 29166007

[ref5] Tsuji Y.; Okazawa K.; Kobayashi Y.; Kageyama H.; Yoshizawa K. Electronic origin of catalytic activity of TiH_2_ for ammonia synthesis. J. Phys. Chem. C 2021, 125, 3948–3960. 10.1021/acs.jpcc.0c10907.

[ref6] Orimo S. I.; Nakamori Y.; Eliseo J. R.; Züttel A.; Jensen C. M. Complex hydrides for hydrogen storage. Chem. Rev. 2007, 107, 4111–4132. 10.1021/cr0501846. 17848101

[ref7] Bogdanović B.; Schwickardi M. Ti-doped alkali metal aluminium hydrides as potential novel reversible hydrogen storage materials. J. Alloys Compd. 1997, 253–254, 1–9. 10.1016/S0925-8388(96)03049-6.

[ref8] Frankcombe T. J. Proposed mechanisms for the catalytic activity of Ti in NaAlH_4_. Chem. Rev. 2012, 112, 2164–2178. 10.1021/cr2001838. 22166103

[ref9] Léon A.; Schild D.; Fichtner M. Chemical state of Ti in sodium alanate doped with TiCl_3_ using X-ray photoelectron spectroscopy. J. Alloys Compd. 2005, 404–406, 766–770. 10.1016/j.jallcom.2004.11.129.

[ref10] Graetz J.; Reilly J. J.; Johnson J.; Ignatov A. Y.; Tyson T. A. X-ray absorption study of Ti-activated sodium aluminum hydride. Appl. Phys. Lett. 2004, 85, 500–502. 10.1063/1.1773614.

[ref11] Zhou C.; Zhang J.; Bowman R. C.; Fang Z. Z. Roles of Ti-based catalysts on magnesium hydride and its hydrogen storage properties. Inorganics 2021, 9, 36 10.3390/inorganics9050036.

[ref12] Hall P. G.; Hope C. J. Adsorption of non-polar gases on iron and titanium. J. Chem. Soc. A 1970, 2003–2008. 10.1039/j19700002003.

[ref13] Kasajima T.; Nishikiori T.; Nohira T.; Ito Y. Thermodynamic evaluation of Ti-H system at medium-range temperatures by molten salt electrochemical technique. J. Electrochem. Soc. 2003, 150, E355 10.1149/1.1580152.

[ref14] San-Martin A.; Manchester F. D. The H-Ti (hydrogen-titanium) system. Bull. Alloy Phase Diagrams 1987, 8, 30–42. 10.1007/BF02868888.

[ref15] Christmann K. Interaction of hydrogen with solid surfaces. Surf. Sci. Rep. 1988, 9, 1–163. 10.1016/0167-5729(88)90009-X.

[ref16] Billeter E.; Terreni J.; Borgschulte A. Hydride formation diminishes CO_2_ reduction rate on palladium. ChemPhysChem 2019, 20, 1398–1403. 10.1002/cphc.201801081. 30561889 PMC6590662

[ref17] Wilde M.; Fukutani K. Penetration mechanisms of surface-adsorbed hydrogen atoms into bulk metals: Experiment and model. Phys. Rev. B 2008, 78, 115411 10.1103/PhysRevB.78.115411.

[ref18] Brown C. C.; Buxbaum R. E. Kinetics of hydrogen absorption in alpha titanium. Metall. Mater. Trans. A 1988, 19, 1425–1427. 10.1007/BF02674016.

[ref19] Wulv H. G.; Fromm E. Hydrogen absorption rate of titanium, lanthanum, iron, nickel, manganese and palladium films with and without ovygen precoverage at 300 K. J. Less-Common Met. 1986, 118, 293–301. 10.1016/0022-5088(86)90180-3.

[ref20] Schlapbach L. Surface Properties and Activation. In Hydrogen in Intermetallic Compounds II, Surface and Dynamic Properties, Applications; Schlapbach L., Ed.; Topics in Applied Physics; Springer, 1992.

[ref21] Hayoz J.; Pillo T.; Bovet M.; Züttel A.; Guthrie S.; Pastore G.; Schlapbach L.; Aebi P. Preparation and characterization of clean, single-crystalline YH_*x*_ films (0≤x≤2.9) on W(110). J. Vac. Sci. Technol., A 2000, 18, 2417–2431. 10.1116/1.1286073.

[ref22] Tsuchiya B.; Oku M.; Sahara R.; Nagata S.; Shikama T.; Kawazoe Y. Electronic structure of the bulk of titanium hydrides fractured in ultrahigh vacuum by XPS surface analysis. J. Surf. Anal. 2008, 14, 424–427.

[ref23] Lundgren E.; Gustafson J.; Mikkelsen A.; Andersen J. N.; Stierle A.; Dosch H.; Todorova M.; Rogal J.; Reuter K.; Scheffler M. Kinetic hindrance during the initial oxidation of Pd(100) at ambient pressures. Phys. Rev. Lett. 2004, 92, 046101 10.1103/PhysRevLett.92.046101. 14995387

[ref24] Borgschulte A.; Westerwaal R. J.; Rector J. H.; Dam B.; Griessen R.; Schoenes J. Effect of the strong metal-support interaction on hydrogen sorption kinetics of Pd-capped switchable mirrors. Phys. Rev. B 2004, 70, 155414 10.1103/PhysRevB.70.155414.

[ref25] Wipf H. Hydrogen in Metals III, Topics in Applied Physics; Springer: Berlin; Vol. 73.

[ref26] Sambalova O.; Borgschulte A. Membrane concept for environmental surface science. J. Alloys Compd. 2018, 742, 518–523. 10.1016/j.jallcom.2018.01.160.

[ref27] Team R. C. R: A Language and Environment for Statistical Computing; R Development Core Team, 2017.

[ref28] Team R. RStudio: Integrated Development for R. RStudio; PBC: Boston, MA, 2020.

[ref29] Biesinger M. C.; Lau L. W.; Gerson A. R.; Smart R. S. C. Resolving surface chemical states in XPS analysis of first row transition metals, oxides and hydroxides: Sc, Ti, V, Cu and Zn. Appl. Surf. Sci. 2010, 257, 887–898. 10.1016/j.apsusc.2010.07.086.

[ref30] Kresse G.; Furthml̈ler J. Efficient iterative schemes for ab initio total-energy calculations using a plane-wave basis set. Phys. Rev. B 1996, 54, 11169 10.1103/PhysRevB.54.11169. 9984901

[ref31] Kresse G.; Furthmüller J. Efficiency of ab-initio total energy calculations for metals and semiconductors using a plane-wave basis set. Comput. Mater. Sci. 1996, 6, 15–50. 10.1016/0927-0256(96)00008-0. 9984901

[ref32] Blöchl P. E. Projector augmented-wave method. Phys. Rev. B 1994, 50, 17953 10.1103/PhysRevB.50.17953. 9976227

[ref33] Kresse G.; Joubert D. From ultrasoft pseudopotentials to the projector augmented-wave method. Phys. Rev. B 1999, 59, 1758 10.1103/PhysRevB.59.1758.

[ref34] Perdew J. P.; Burke K.; Ernzerhof M. Generalized gradient approximation made simple. Phys. Rev. Lett. 1996, 77, 3865 10.1103/PhysRevLett.77.3865. 10062328

[ref35] Henkelman G.; Uberuaga B. P.; Jónsson H. A climbing image nudged elastic band method for finding saddle points and minimum energy paths. J. Chem. Phys. 2000, 113, 9901–9904. 10.1063/1.1329672.

[ref36] Krozer A.; Fischer A.; Schlapbach L. Experimental study of the valence-band region of Mg-Pd and Ba-Pd interfaces with and without hydrogen and of Mg and Ba hydrides. Phys. Rev. B 1996, 53, 13808–13816. 10.1103/PhysRevB.53.13808. 9983135

[ref37] Delmelle R.; Probst B.; Alberto R.; Züttel A.; Bleiner D.; Borgschulte A. Closing the pressure gap in x-ray photoelectron spectroscopy by membrane hydrogenation. Rev. Sci. Instrum. 2015, 86, 053104 10.1063/1.4921353. 26026511

[ref38] Lamartine B. C.; Haas T. W.; Solomon J. S. Characterization of TiH_*x*_ and TiD_0.9_ surfaces: AES, ELS, SIMS and XPS Studies. Appl. Surf. Sci. 1980, 4, 537–555. 10.1016/0378-5963(80)90097-5.

[ref39] Kihn Y.; Mirguet C.; Calmels L. EELS studies of Ti-bearing materials and ab initio calculations. J. Electron Spectrosc. Relat. Phenom. 2005, 143, 117–127. 10.1016/j.elspec.2004.02.170.

[ref40] Wadell C.; Syrenova S.; Langhammer C. Plasmonic hydrogen sensing with nanostructured metal hydrides. ACS Nano 2014, 8, 11925–11940. 10.1021/nn505804f. 25427244

[ref41] Baldi A.; Narayan T.; Koh A.; Dionne J. A. In situ detection of hydrogen-induced phase transitions in individual palladium nanocrystals. Nat. Mater. 2014, 13, 1143–1148. 10.1038/nmat4086. 25194700

[ref42] Werner W. S. M. Analysis of reflection electron energy loss spectra (REELS) for determination of the dielectric function of solids: Fe, Co, Ni. Surf. Sci. 2007, 601, 2125–2138. 10.1016/j.susc.2007.03.001.

[ref43] Werner W. S. M. Electron transport in solids for quantitative surface analysis. Surf. Interface Anal. 2001, 31, 141–176. 10.1002/sia.973.

[ref44] Brüesch P. Phonons: Theory and Experiments II: Experiments and Interpretation of Experimental Results; Springer: Heidelberg, 1986.

[ref45] Herley P. J.; Jones W.; Sparrow T. G.; Williams B. G. Plasmon spectra of light-metal hydrides. Mater. Lett. 1987, 5, 333–336. 10.1016/0167-577X(87)90122-4.

[ref46] Wang W. E. Thermodynamic evaluation of the titanium-hydrogen system. J. Alloys Compd. 1996, 238, 6–12. 10.1016/0925-8388(96)02264-5.

[ref47] Wagner C.; Joshi A. The auger parameter, its utility and advantages: a review. J. Electron Spectrosc. Relat. Phenom. 1988, 47, 283–313. 10.1016/0368-2048(88)85018-7.

[ref48] Moretti G. Auger parameter and Wagner plot in the characterization of chemical states by X-ray photoelectron spectroscopy: A review. J. Electron Spectrosc. Relat. Phenom. 1998, 95, 95–144. 10.1016/S0368-2048(98)00249-7.

[ref49] Crank J. The Mathematics of Diffusion; Oxford University Press, 1957.

[ref50] Bracconi P.; Lässer R. Investigation of titanium and titanium hydride by AES and EELS. Appl. Surf. Sci. 1987, 28, 204–214. 10.1016/0169-4332(87)90122-X.

[ref51] Kim Y.-J.; Tao R.; Klie R. F.; Seidman D. N. Direct atomic-scale imaging of hydrogen and oxygen interstitials in pure niobium using atom-probe tomography and aberration-corrected scanning transmission electron microscopy. ACS Nano 2013, 7, 732–739. 10.1021/nn305029b. 23259811

[ref52] Dantzer P. High temperature thermodynamics of H2 and D2 in titanium, and in dilute titanium oxygen solid solutions. J. Phys. Chem. Solids 1983, 44, 913–923. 10.1016/0022-3697(83)90130-0.

[ref53] Hammer B.; Nørskov J. Why gold is the noblest of all the metals. Nature 1995, 376, 238–240. 10.1038/376238a0.

[ref54] Hammer B.; Nørskov J. Impact of Surface Science on Catalysis. In Advances in Catalysis; Academic Press, 2000; Vol. 45, pp 71–129.

[ref55] Greeley J.; Mavrikakis M. Alloy catalysts designed from first principles. Nat. Mater. 2004, 3, 810–815. 10.1038/nmat1223. 15502837

[ref56] Borgschulte A.; Westerwaal R.; Rector J.; Schreuders H.; Dam B.; Griessen R. Catalytic activity of noble metals promoting hydrogen uptake. J. Catal. 2006, 239, 263–271. 10.1016/j.jcat.2006.01.031.

[ref57] Wedler G.; Strothenk H. Elektrische und kalorimetrische Messungen am System Titan/Wasserstoff bei 273 °K. Z. Phys. Chem. 1966, 48, 86–101. 10.1524/zpch.1966.48.1_2.086.

[ref58] Cremaschi P.; Whitten J. L. Chemisorption of hydrogen on titanium: Embedding theory and comparisons with small clusters. Surf. Sci. 1981, 112, 343–358. 10.1016/0039-6028(81)90379-4.

[ref59] Arita M.; Shimizu K.; Ichinose Y. Thermodynamics of the Ti-H system. Metall. Mater. Trans. A 1982, 13, 1329–1336. 10.1007/BF02642869.

[ref60] Zabel H.; Peisl H. Sample-shape-dependent phase transition of hydrogen in niobium. Phys. Rev. Lett. 1979, 42, 511–514. 10.1103/PhysRevLett.42.511.

[ref61] Spatschek R.; Gobbi G.; Hüter C.; Chakrabarty A.; Aydin U.; Brinckmann S.; Neugebauer J. Scale bridging description of coherent phase equilibria in the presence of surfaces and interfaces. Phys. Rev. B 2016, 94, 134106 10.1103/PhysRevB.94.134106.

[ref62] Bustard L. D.; Cotts R. M.; Seymour E. F. W. Determination of the hydrogen diffusion mechanism in γ-titanium hydride using nuclear magnetic resonance. Phys. Rev. B 1980, 22, 12–20. 10.1103/PhysRevB.22.12.

[ref63] Fukai Y. The Metal-Hydrogen System; Springer-Verlag: Berlin, Heidelberg, 1993.

[ref64] Nishimura C.; Komaki M.; Amano M. Hydrogen permeation through magnesium. J. Alloys Compd. 1999, 293–295, 329–333. 10.1016/S0925-8388(99)00373-4.

[ref65] Čermák J.; Král L. Hydrogen diffusion in Mg-H and Mg-Ni-H alloys. Acta Mater. 2008, 56, 2677–2686. 10.1016/j.actamat.2008.02.003.

[ref66] Benvenuti C.; Chiggiato P.; Cicoira F.; L’Aminot Y. Nonevaporable getter films for ultrahigh vacuum applications. J. Vac. Sci. Technol., A 1998, 16, 148–154. 10.1116/1.580963.

[ref67] Fokin V.; Malov Y.; Fokina E.; Troitskaya S.; Shilkin S. Investigation of interactions in the TiH2-O2 system. Int. J. Hydrogen Energy 1995, 20, 387–389. 10.1016/0360-3199(94)00075-B.

[ref68] Ye T.; Park S.; Lu Y.; Li J.; Sasase M.; Kitano M.; Tada T.; Hosono H. Vacancy-enabled N2 activation for ammonia synthesis on an Ni-loaded catalyst. Nature 2020, 583, 391–395. 10.1038/s41586-020-2464-9. 32669696

[ref69] Jonker B. T.; Morar J. F.; Park R. L. Surface states and oxygen chemisorption on Ti(0001). Phys. Rev. B 1981, 24, 2951–2957. 10.1103/PhysRevB.24.2951.

[ref70] Didziulis S. V.; Butcher K. D.; Perry S. S. Small Cluster Models of the Surface Electronic Structure and Bonding Properties of Titanium Carbide, Vanadium Carbide, and Titanium Nitride. Inorg. Chem. 2003, 42, 7766–7781. 10.1021/ic030140k. 14632492

[ref71] Borgschulte A.; Bielmann M.; Züttel A.; Barkhordarian G.; Dornheim M.; Bormann R. Hydrogen dissociation on oxide covered MgH2 by catalytically active vacancies. Appl. Surf. Sci. 2008, 254, 2377–2384. 10.1016/j.apsusc.2007.09.069.

[ref72] Du A. J.; Smith S. C.; Lu G. Q. Role of charge in destabilizing AlH_4_ and BH_4_ complex anions for hydrogen storage applications: Ab initio density functional calculations. Phys. Rev. B 2006, 74, 193405 10.1103/PhysRevB.74.193405.

[ref73] Du A. J.; Smith S. C.; Lu G. Q. Vacancy mediated desorption of hydrogen from a sodium alanate surface: An ab initio spin-polarized study. Appl. Phys. Lett. 2007, 90, 143119 10.1063/1.2721127.

[ref74] Streukens G.; Bogdanović B.; Felderhoff M.; Schüth F. Dependence of dissociation pressure upon doping level of Ti-doped sodium alanate—a possibility for “thermodynamic tailoring” of the system. Phys. Chem. Chem. Phys. 2006, 8, 2889–2892. 10.1039/B603268K. 16775644

[ref75] Brinks H. W.; Hauback B. C.; Srinivasan S. S.; Jensen C. M. Synchrotron X-ray studies of Al1-yTiy formation and re-hydriding inhibition in Ti-enhanced NaAlH4. J. Phys. Chem. B 2005, 109, 15780–15785. 10.1021/jp051031p. 16853003

[ref76] Wang P.; Kang X.-D.; Cheng H.-M. Exploration of the nature of active Ti species in metallic Ti-doped NaAlH4. J. Phys. Chem. B 2005, 109, 20131–20136. 10.1021/jp053152v. 16853602

[ref77] Léon A.; Kircher O.; Rothe J.; Fichtner M. Chemical state and local structure around titanium atoms in NaAlH_4_ doped with TiCl_3_ using X-ray absorption spectroscopy. J. Phys. Chem. B 2004, 108, 16372–16376. 10.1021/jp048615w. 16471663

